# Synthesis and biological evaluation of new 3(2*H*)-pyridazinone derivatives as non-toxic anti-proliferative compounds against human colon carcinoma HCT116 cells

**DOI:** 10.1080/14756366.2020.1755670

**Published:** 2020-04-22

**Authors:** Zeynep Özdemir, Semra Utku, Bijo Mathew, Simone Carradori, Giustino Orlando, Simonetta Di Simone, Mehmet Abdullah Alagöz, Azime Berna Özçelik, Mehtap Uysal, Claudio Ferrante

**Affiliations:** a Department of Pharmaceutical Chemistry, İnönü University, Malatya, Turkey; b Department of Pharmaceutical Chemistry, Mersin University, Mersin, Turkey; c Department of Pharmaceutical Chemistry, Division of Drug Design and Medicinal Chemistry Research Lab, Ahalia School of Pharmacy, Palakkad, India; d Department of Pharmacy, “G. d’Annunzio” University of Chieti-Pescara, Chieti, Italy; e Department of Pharmaceutical Chemistry, Gazi University, Ankara, Turkey; f Department of Pharmaceutical Chemistry, Erzincan Binali Yıldırım University, Erzincan, Turkey

**Keywords:** Serotonin, anti-proliferative agents, pyridazinone, kynurenic acid, HCT116, wound healing, *Artemia salina* lethality test

## Abstract

Novel 3(2*H*)-pyridazinone derivatives were designed, synthesised in satisfactory yields and evaluated in different experimental assays to assess their preliminary toxicity *in vivo* and anti-proliferative effects against HCT116 cell lines *in vitro*. *Artemia salina* lethality test provided LC_50_ values >100 µg/mL for all compounds. Successive assays revealed that some compounds were endowed with a promising anti-proliferative effect against HCT116 cells, alone or stimulated by serotonin as a pro-inflammatory factor in order to mimick an inflamed model *in vivo* of cancer cell microenvironment. Moreover, the kinurenic acid level after treatment with these newly synthesised compounds was monitored as a marker of anti-proliferation in colon carcinoma models. The IC_50_ values obtained for the best-in-class compounds were comparable to that of daunorubicin as a reference drug. Conversely, these compounds were not able to counteract the spontaneous migration of human cancer HCT116 cell line in the wound healing paradigm.

## 1. Introduction

Cancer consists of an uncontrolled proliferation of cells in different tissues and organs; it is a disease whose clinical appearance, treatment and approach are different from each other. Cancer is a major global health problem and it is currently the second leading cause of death in the world being expected to surpass cardiovascular diseases in the next few years^1^^,^^2^. Many factors, from bacteria to viruses, from radiation to heredity, from environmental factors to nutritional habits and chemicals, are accused of cancer formation. In the data announced by the World Health Organisation (WHO), approximately 18 million people were diagnosed with cancer in 2018, and around 10 million people died from cancer. According to data of Global Cancer Obervatory (GLOBOCAN), the most common types are lung (2.1 million), breast (2.09 million), colorectal (1.8 million), prostate (1.3 million), stomach (1 million) cancer. According to cancer-related deaths, lung (1.8 million), colorectal (881 thousand), stomach (783 thousand), liver (782 thousand) and breast (627 thousand) are listed. Colorectal carcinomas (CRC) are one of the most common types of cancer in the world that cause death. CRC metastases account for 40–50% of recently diagnosed cases and are correlated with high morbidity^3^^,^^4^.

**Figure 1.**
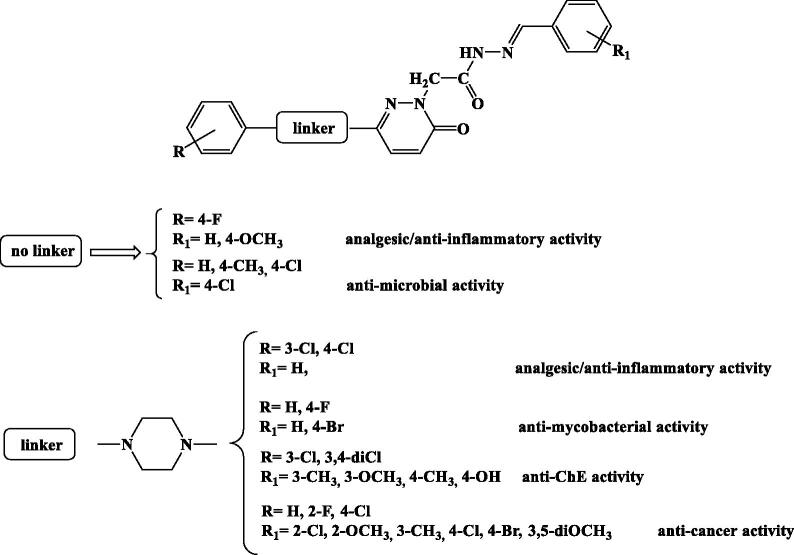
Structure-activity relationships (SARs) within the 3(2*H*)-pyridazinone derivatives reported in the literature.

In medicinal chemistry pyridazinones have been the subject of intensive synthetic investigations, because they possess a wide spectrum of pharmacological activities and gained importance in recent years^5^. A number of compounds such as zardaverine/imidazole, bemoradan, indolindan, pimobendan are examples of pyridazinones that are biologically active. Literature survey revealed that substituted pyridazinones have reported to possess pharmacological activities, which can be rationalised in the SAR study reported in Figure 1 ^6–12^. There are also compounds which were shown to have anti-cancer or cytostatic activity in the literature against HEP3B (liver cancer cells), HCT116 (colon cancer cells), SH-SY5Y (neuroblastoma cells) and promising selectivity index with respect to human fibroblasts^13–16^. These results suggest that pyridazinone compounds may be useful in cancer chemotherapy, depending on the type of cancer, and that derivatives bearing different substituents may exhibit varying degrees of cytotoxic effect.

Pursuing our efforts to discover novel anti-cancer compounds^17–19^ and with the aim of enlarging the SAR knowledge within this chemical scaffold, we designed fifteen new 3(2*H*)-pyridazinones investigating the anti-proliferative effects against the human HCT116 cell line, their toxicity in the *Artemia salina* lethality assay *in vivo*, the HCT116 viability after serotonin challenging and compound treatment, the release of kynurenic acid after compound treatment and, lastly, the capability to limit the spontaneous migration of HCT116 cells in the wound healing paradigm.

## 2. Experimental protocols

The fine chemicals and all solvents used in this study were purchased locally from Merck (Darmstadt, Germany) and Aldrich Chemical Co. (Steinheim, Germany).

### 2.1. Chemical studies

Melting points of the compounds were determined on Electrothermal 9200 melting points apparatus (Southent, Great Britain) and the values given are uncorrected. The IR spectra of the compounds were recorded on a Bruker Vector 22 IR Spectrophotometer (Bruker Analytische Messtechnik, Karlsruhe, Germany). The ^1^H-NMR and 13C-NMR spectra of the compounds were recorded on a Bruker 400 MHz-NMR Spectrometer (Rheinstetten, Karlsruhe, Germany) using tetramethylsilane as an internal standard. All the chemical shifts were recorded as *δ* (ppm). The mass spectra (HRMS) of the compounds were recorded on Waters Acquity Ultra Performance Liquid Chromatograpy Micromass which combined LCT PremierTM XE UPLC/MS TOFF spectrophotometer (Waters Corp, Milford, USA) by ESI + and ESI– techniques.

#### 2.1.1. Synthesis of 4-(3-fluoro-4-methoxyphenyl)-4-oxobutanoic acid (I)

A mixture of 0.275 mol aluminium chloride, 20 mL carbon disulphide and 0.25 mol succinic anhydride was added portionwise in standard conditions to a mixture of 0.25 mol 2-fluoroanisole and 50 mL of carbon disulphide. Then, the mixture was refluxed for 4 h at 40–50 °C. After cooling, the residue was poured onto ice water and the precipitate was collected, dried and recrystallized from water. M.P.: 164 °C. (Lit: M.P. 168 °C). Yield: 78%, C_11_H_12_FO_4_ Calcd.: 226.0720, Found: 227.0724. NMR spectra are in accordance with literature data.

#### 2.1.2. Synthesis of 6-(3-fluoro-4-methoxyphenyl)-4,5-dihydro-3(2H)-pyridazinone (II)

0.01 Mol of 4-(3-fluoro-4-methoxyphenyl)-4-oxobutanoic acid and 0.015 mol of hydrazine hydrate (0.85 mL; 55%) in 30 mL of ethanol were refluxed for 4 h. The reaction mixture was cooled and the precipitate thus formed was collected by filtration, dried, crystallised from ethanol. M.P.: 182 °C. (Lit: M.P. 180–182 °C). Yield: 58%, C_11_H_12_FN_2_O_2_ Calcd.: 223.2270. Found: 223.2854. NMR spectra are in accordance with literature data.

#### 2.1.3. Synthesis of 6-(3-fluoro-4-methoxyphenyl)-3(2H)-pyridazinone (III)

A solution of 0.043 mol of bromine in 25 mL of glacial acetic acid was added dropwise to a solution of 0.039 mol of 6-(3-fluoro-4-methoxyphenyl)-4,5-dihydro-3(2*H*)-pyridazinone (**II**) in 100 mL of glacial acetic acid at 60–70 °C. Then, the reaction mixture was refluxed for 3 h. After cooling to 5 °C, it was poured into ice water and converted to free base with ammonium hydroxide. The precipitate was collected by filtration, washed with cold water until neutral, dried, and crystallised from ethanol-water mixture. M.P.: 221 °C. (Lit: M.P. 220–222 °C). Yield: 76%, C_11_H_10_FN_2_O_2_ Calcd.: 221.0726, Found: 221.0721. NMR spectra are in accordance with literature data.

#### 2.1.4. Synthesis of ethyl 6-(3-fluoro-4-methoxyphenyl)-3(2H)-pyridazinone-2-yl-acetate (IV)

A mixture of required 6-substituted-3(2*H*)-pyridazinones (**III**) (0.01 mol), ethyl 3-bromo-acetate (0.02 mol) and potassium carbonate (0.02 mol) in acetone (40 mL) was refluxed overnight. After the mixture was cooled, the organic salts were filtered off, the solvent evaporated, and the residue was purified by recrystallization from ethanol to give the ester. M.P.: 126 °C. Yield: 69%, C_15_H_16_FN_2_O_4_ Calcd.: 307.1094, Found: 307.1074. ^1^H-NMR (400 MHz) (DMSO-d_6_): *δ* 8.09 (d, 1H, pyridazinone H_5_), 7.05–7.82 (m, 3H, phenyl H_2_, H_5_, H_6_), 7.31 (t, 1H, pyridazinone H_4_), 4.88 (s, 2H, CH_2_COOCH_2_CH_3_), 4.10 (q, 2H, CH_2_COOCH_2_CH_3_), 3.91 (s, 3H, OCH_3_), 1.22 (t, 3H, CH_2_COOCH_2_CH_3_).

#### 2.1.5. Synthesis of 6-(3-fluoro-4-methoxyphenyl)-3(2H)-pyridazinone-2-yl-acetohydrazide (V)

To the ethanolic solution of ethyl 6-substituted-3(2*H*)-pyridazinone-2-yl-acetate (**IV**) (25 mL, 0.01 mol) hydrazine hydrate (99%) (3 mL) was added and stirred for 3 h at room temperature. The obtained precipitate was filtered off, washed with water, dried and recrystallized from ethanol. M.P.: 206 °C. Yield: 76%, C_13_H_13_FN_4_O_3_ Calcd.: 293.1050, Found: 293.1060.

#### 2.1.6. Synthesis of substituted/nonsubstituted benzalhydrazone derivatives of 6-(3-fluoro-4-methoxyphenyl)-3(2H)-pyridazinone-2-yl-acetohydrazide (via-o)

A mixture of 6-(3-fluoro-4-methoxyphenyl)-3(2*H*)-pyridazinone-2-yl-acetohydrazide **V** (0.01 mol) and appropriate benzaldehyde (0.01 mol) was refluxed in ethanol (15 mL) for 6 h. Then, the mixture was poured into ice water. The formed precipitate was recrystallized from the appropriate solvent. The compounds were identified by IR, ^1^H-NMR, 13C-NMR and mass spectra. All spectral data of the compounds were in accordance with the assigned structures as shown below.

##### 2.1.6.1. N’-benzylidene-2-(3-(3-fluoro-4-methoxyphenyl)-6-oxopyridazin-1(6H)-yl)acetohydrazide (VIa)

White crystals; yield: 80%; M.P.: 132 °C; IR (ν cm^−1^, ATR): 1698 (C=O; hydrazone), 1648 (C=O; pyridazinone ring), 1587 (C=N); ^1^H-NMR (DMSO-d_6_, 300 MHz): *δ* 3.90 (3H; s; CH_3_O), 5.30 (2H; s; –N–CH_2_–C=O), 7.09 (1H; d; pyridazinone H^5^), 7.27 (1H; d; pyridazinone H^4^), 7.11–8.14 (8H; m; phenyl protons), 8.24 (1H; s; –N=CH–), 11.76 (1H; s; –NH–N). 13C-NMR (DMSO-d_6_, 300 MHz): *δ* 53.9 (1C; CH_3_O), 56.7 (1C; –N–CH_2_–C=O), 113.4 (1C; =CH), 113.8 (1C; pyridazinone C^5^), 114.5 (1C; phenyl C^4^), 127.3 (2C; phenyl C^3,^5), 129.1 (2C; phenyl C^2,6^), 134.3 (1C; pyridazinone C^4^), 142.8 (2C; 3-fluoro-4-methoxyphenyl C^2,6^), 144.5 (1C; phenyl C^1^), 147.6 (2C; 3-fluoro-4-methoxyphenyl C^3,5^), 148.6 (1C; 3-fluoro-4-methoxyphenyl C^1^), 151.2 (1C; pyridazinone C^6^), 159.3 (1C; 3-fluoro-4-methoxyphenyl C^4^), 163.5 (1C; CH_2_–N–C=O), 168.2 (1C; pyridazinone C^3^); C_20_H_17_FN_4_O_3_ MS (ESI+) Calcd.: 381.1348, Found: *m*/*z* 381.1348 (M^+^; 100.0%).

##### 2.1.6.2. N’-(4-fluorobenzylidene)-2–(3-(3-fluoro-4-methoxyphenyl)-6-oxopyridazin-1(6H)-yl)acetohydrazide (VIb)

White crystals; yield: 83%; M.P.: 238 °C; IR (ν cm^−1^, ATR): 1693 (C=O; hydrazone), 1652 (C=O; pyridazinone ring), 1514 (C=N); ^1^H-NMR (DMSO-d_6_, 300 MHz): *δ* 3.90 (3H; s; CH_3_O), 5.30 (2H; s; –N–CH_2_–C=O), 7.08 (1H; d; pyridazinone H^5^), 7.11 (1H; d; pyridazinone H^4^), 7.27–7.82 (7H; m; phenyl protons), 8.10 (1H; s; –N=CH–), 11.76 (1H; s; –NH–N); ^13^C-NMR (DMSO-d_6_, 300 MHz): *δ* 53.7 (1C; CH_3_O), 56.7 (1C; –N–CH_2_–C=O), 113.4 (1C; =CH), 113.6 (1C; pyridazinone C^5^), 114.3 (2C; 4-fluorophenyl C^3,5^), 116.3 (2C; 4-fluorophenyl C^2,6^), 127.5 (1C; pyridazinone C^4^), 129.5 (2C; 3-fluoro-4-methoxyphenyl C^2,6^), 129.6 (1C; 4-fluorophenyl C^1^), 131.4 (2C; 3-fluoro-4-methoxyphenyl C^3,5^), 142.8 (1C; 3-fluoro-4-methoxyphenyl C^1^), 148.6 (1C; pyridazinone C^6^), 152.9 (1C; 4-fluorophenyl C^4^), 159.3 (1C; 3-fluoro-4-methoxyphenyl C^4^), 164.3 (1C; CH_2_–N–C=O), 168.3 (1C; pyridazinone C^3^); C_20_H_17_F_2_N_4_O_3_ MS (ESI+) Calcd.: 399.1269, Found: *m*/*z* 399.1285 (M^+^; 100.0%).

##### 2.1.6.3. N’-(4-trifluoromethylbenzylidene)-2–(3-(3-fluoro-4-methoxyphenyl)-6-oxopyridazin-1(6H)-yl)acetohydrazide (VIc)

White crystals; yield: 84%; M.P.: 252 °C; IR (ν cm^−1^, ATR): 1704 (C=O; hydrazone), 1699 (C=O; pyridazinone ring), 1587 (C=N); ^1^H-NMR (DMSO-d_6_, 300 MHz): *δ* 3.90 (3H; s; CH_3_O), 5.33 (2H; s; –N–CH_2_–C=O), 7.10 (1H; d; pyridazinone H^5^), 7.26 (1H; d; pyridazinone H^4^), 7.12–7.97 (7H; m; phenyl protons), 8.14 (1H; s; –N=CH–), 11.96 (1H; s; –NH–N); ^13^C-NMR (DMSO-d_6_, 300 MHz): *δ* 53.9 (1C; CH_3_O), 56.7 (1C; –N–CH_2_–C=O), 113.4 (1C; CF_3_), 113.6 (1C; =CH), 114.3 (1C; pyridazinone C^5^), 114.5 (2C; 4-fluorophenyl C^3,5^), 126.0 (2C; 4-fluorophenyl C^2,6^), 127.5 (1C; pyridazinone C^4^), 128.1 (2C; 3-fluoro-4-methoxyphenyl C^2,6^), 131.4 (1C; 4-fluorophenyl C^1^), 138.3 (2C; 3-fluoro-4-methoxyphenyl C^3,5^), 142.8 (1C; 3-fluoro-4-methoxyphenyl C^1^), 151.2 (1C; pyridazinone C^6^), 152.9 (1C; 4-fluorophenyl C^4^), 159.4 (1C; 3-fluoro-4-methoxyphenyl C^4^), 163.8 (1C; CH_2_–N–C=O), 168.5 (1C; pyridazinone C^3^); C_21_H_17_F_4_N_4_O_3_ MS (ESI+) Calcd.: 449.1237, Found: *m*/*z* 449.1237 (M^+^; 100.0%).

##### 2.1.6.4. N’-(2-fluorobenzylidene)-2–(3-(3-fluoro-4-methoxyphenyl)-6-oxopyridazin-1(6H)-yl)acetohydrazide (VId)

White crystals; yield: 68%; M.P.: 222 °C; IR (ν cm^−1^, ATR): 1696 (C=O; hydrazone), 1676 (C=O; pyridazinone ring), 1596 (C=N); ^1^H-NMR (DMSO-d_6_, 300 MHz): *δ* 3.87 (3H; s; CH_3_O), 5.34 (2H; s; –N–CH_2_–C=O), 7.09 (1H; d; pyridazinone H^5^), 7.12 (1H; d; pyridazinone H^4^), 7.10–8.26 (7H; m; phenyl protons), 8.40 (1H; s; –N=CH–), 12.00 (1H; s; –NH–N); ^13^C-NMR (DMSO-d_6_, 300 MHz): *δ* 53.9 (1C; CH_3_O), 56.6 (1C; –N–CH_2_–C=O), 113.5 (1C; CF_3_), 113.7 (1C; =CH), 114.4 (1C; pyridazinone C^5^), 127.4 (1C; 2-trifluoromethylphenyl C^5^), 130.0 (1C; 2-trifluoromethylphenyl C^4^), 130.5 (1C; 2-trifluoromethylphenyl C^6^), 132.2 (1C; 2-trifluoromethylphenyl C^3^), 133.3 (1C; pyridazinone C^4^), 139.8 (1C; 3-fluoro-4-methoxyphenyl C^6^), 142.7 (1C; 3-fluoro-4-methoxyphenyl C^5^), 142.9 (1C; 2-trifluoromethylphenyl C^1^), 148.6 (1C; 3-fluoro-4-methoxyphenyl C^2^), 148.6 (1C; 3-fluoro-4-methoxyphenyl C^1^), 151.2 (1C; 2-trifluoromethylphenyl C^2^), 152.9 (1C; pyridazinone C^6^), 159.3 (1C; 3-fluoro-4-methoxyphenyl C^3^), 159.4 (1C; 3-fluoro-4-methoxyphenyl C^4^), 163.8 (1C; CH_2_–N–C=O), 168.5 (1C; pyridazinone C^3^); C_21_H_17_F_4_N_4_O_3_ MS (ESI+) Calcd.: 449.1237, Found: *m*/*z* 449.1258 (M^+^; 100.0%).

##### 2.1.6.5. N’-(2-methylbenzylidene)-2–(3-(3-fluoro-4-methoxyphenyl)-6-oxopyridazin-1(6H)-yl)acetohydrazide (VIe)

White crystals; yield: 96%; M.P.: 210 °C; IR (ν cm^−1^, ATR): 1700 (C=O; hydrazone), 1640 (C=O; pyridazinone ring), 1588 (C=N); ^1^H-NMR (DMSO-d_6_, 300 MHz): *δ* 2.45 (3H; s; CH_3_), 3.90 (3H; s; CH_3_O), 5.29 (2H; s; –N–CH_2_–C=O), 7.08 (1H; d; pyridazinone H^5^), 7.11 (1H; d; pyridazinone H^4^), 7.09–8.13 (7H; m; phenyl protons), 8.31 (1H; s; –N=CH–), 11.69 (1H; s; –NH–N); ^13^C-NMR (DMSO-d_6_, 300 MHz): *δ* 20.1 (1C; CH_3_), 53.9 (1C; CH_3_O), 56.5 (1C; –N–CH_2_–C=O), 113.6 (1C; =CH), 114.3 (1C; pyridazinone C^5^), 126.5 (1C; 2-methylphenyl C^5^), 126.6 (1C; 2-methylphenyl C^4^), 127.1 (1C; 2-methylphenyl C^6^), 130.0 (1C; 2-methylphenyl C^3^), 131.3 (1C; pyridazinone C^4^), 131.4 (1C; 3-fluoro-4-methoxyphenyl C^6^), 132.3 (1C; 3-fluoro-4-methoxyphenyl C^5^), 137.1 (1C; 2-methylphenyl C^1^), 142.8 (1C; 2-methylphenyl C^2^), 143.9 (1C; 3-fluoro-4-methoxyphenyl C^2^), 151.2 (1C; 3-fluoro-4-methoxyphenyl C^1^), 152.9 (1C; pyridazinone C^6^), 159.3 (1C; 3-fluoro-4-methoxyphenyl C^3^), 159.4 (1C; 3-fluoro-4-methoxyphenyl C^4^), 163.4 (1C; CH_2_–N–C=O), 168.1 (1C; pyridazinone C^3^); C_21_H_17_F_4_N_4_O_3_ MS (ESI+) Calcd.: 449.1237, Found: *m*/*z* 449.1258 (M^+^; 100.0%).

##### 2.1.6.6. N’-(2-methoxybenzylidene)-2–(3-(3-fluoro-4-methoxyphenyl)-6-oxopyridazin-1(6H)-yl)acetohydrazide (VIf)

White crystals; yield: 80%; M.P.: 214 °C; IR (ν cm^−1^, ATR): 1693 (C=O; hydrazone), 1649 (C=O; pyridazinone ring), 1589 (C=N); ^1^H-NMR (DMSO-d_6_, 300 MHz): *δ* 3.86 (3H; s; CH_3_O), 3.90 (3H; s; CH_3_O), 5.29 (2H; s; –N–CH_2_–C=O), 7.00 (1H; d; pyridazinone H^5^), 7.09 (1H; d; pyridazinone H^4^), 7.08–8.13 (7H; m; phenyl protons), 8.38 (1H; s; –N=CH–), 11.71 (1H; s; –NH–N); ^13^C-NMR (DMSO-d_6_, 300 MHz): *δ* 53.9 (2C; CH_3_O), 56.7 (1C; –N–CH_2_–C=O), 113.6 (1C; =CH), 114.5 (1C; pyridazinone C^5^), 121.1 (1C; 2-methoxyphenyl C^5^), 122.3 (1C; 2-methoxyphenyl C^4^), 126.0 (1C; 2-methoxyphenyl C^6^), 127.5 (1C; 2-methoxyphenyl C^3^), 131.4 (1C; pyridazinone C^4^), 132.1 (1C; 3-fluoro-4-methoxyphenyl C^6^), 140.1 (1C; 3-fluoro-4-methoxyphenyl C^5^), 140.2 (1C; 2-methoxyphenyl C^1^), 142.8 (1C; 3-fluoro-4-methoxyphenyl C^2^), 148.6 (1C; 3-fluoro-4-methoxyphenyl C^1^), 151.2 (1C; 2-methoxyphenyl C^2^), 152.9 (1C; pyridazinone C^6^), 158.1 (1C; 3-fluoro-4-methoxyphenyl C^3^), 159.3 (1C; 3-fluoro-4-methoxyphenyl C^4^), 159.4 (1C; CH_2_–N–C=O), 168.1 (1C; pyridazinone C^3^); C_21_H_20_FN_4_O_4_ MS (ESI+) Calcd.: 411.1469, Found: *m*/*z* 411.1468 (M^+^; 100.0%).

##### 2.1.6.7. N’-(4-methylbenzylidene)-2–(3-(3-fluoro-4-methoxyphenyl)-6-oxopyridazin-1(6H)-yl)acetohydrazide (VIg)

White crystals; yield: 74%; M.P.: 220 °C; IR (ν cm^−1^, ATR): 1696 (C=O; hydrazone), 1656 (C=O; pyridazinone ring), 1586 (C=N); ^1^H-NMR (DMSO-d_6_, 300 MHz): *δ* 2.34 (3H; s; CH_3_), 3.90 (3H; s; CH_3_O), 5.28 (2H; s; –N–CH_2_–C=O), 7.07 (1H; d; pyridazinone H^5^), 7.10 (1H; d; pyridazinone H^4^), 7.08–8.10 (7H; m; phenyl protons), 8.13 (1H; s; –N=CH–), 11.69 (1H; s; –NH–N); ^13^C-NMR (DMSO-d_6_, 300 MHz): *δ* 20.1 (1C; CH_3_), 53.9 (1C; CH_3_O), 56.5 (1C; –N–CH_2_–C=O), 113.6 (1C; =CH), 114.3 (1C; pyridazinone C^5^), 126.5 (1C; 4-methylphenyl C^5^), 126.6 (1C; 4-methylphenyl C^4^), 127.1 (1C; 4-methylphenyl C^6^), 130.0 (1C; 4-methylphenyl C^3^), 131.3 (1C; pyridazinone C^4^), 131.4 (1C; 3-fluoro-4-methoxyphenyl C^6^), 132.3 (1C; 3-fluoro-4-methoxyphenyl C^5^), 137.1 (1C; 4-methylphenyl C^1^), 142.8 (1C; 4-methylphenyl C^2^), 143.9 (1C; 3-fluoro-4-methoxyphenyl C^2^), 151.2 (1C; 3-fluoro-4-methoxyphenyl C^1^), 152.9 (1C; pyridazinone C^6^), 159.3 (1C; 3-fluoro-4-methoxyphenyl C^3^), 159.4 (1C; 3-fluoro-4-methoxyphenyl C^4^), 163.4 (1C; CH_2_–N–C=O), 168.1 (1C; pyridazinone C^3^); C_21_H_20_FN_4_O_3_ MS (ESI+) Calcd.: 395.1519, Found: *m*/*z* 395.1513 (M^+^; 100.0%).

##### 2.1.6.8. N’-(4-methoxybenzylidene)-2–(3-(3-fluoro-4-methoxyphenyl)-6-oxopyridazin-1(6H)-yl)acetohydrazide (VIh)

White crystals; yield: 85%; M.P.: 207 °C; IR (ν cm^−1^, ATR): 1687 (C=O; hydrazone), 1662 (C=O; pyridazinone ring), 1599 (C=N); ^1^H-NMR (DMSO-d_6_, 300 MHz): *δ* 3.81 (3H; s; CH_3_O), 3.90 (3H; s; CH_3_O), 5.28 (2H; s; –N–CH_2_–C=O), 7.00 (1H; d; pyridazinone H^5^), 7.02 (1H; d; pyridazinone H^4^), 7.03–7.98 (7H; m; phenyl protons), 8.11 (1H; s; –N=CH–), 11.63 (1H; s; –NH–N); ^13^C-NMR (DMSO-d_6_, 300 MHz): *δ* 53.9 (1C; CH_3_O), 54.1 (1C; CH_3_O), 56.7 (1C; –N–CH_2_–C=O), 113.4 (1C; =CH), 113.6 (1C; pyridazinone C^5^), 113.6 (1C; 4-methoxyphenyl C^3^), 113.8 (1C; 4-methoxyphenyl C^5^), 114.3 (1C; 4-methoxyphenyl C^2^), 114.5 (1C; 4-methoxyphenyl C^6^), 126.9 (1C; pyridazinone C^4^), 127.6 (1C; 3-fluoro-4-methoxyphenyl C^2^), 127.6 (1C; 3-fluoro-4-methoxyphenyl C^6^), 129.1 (1C; 4-methoxyphenyl C^1^), 142.8 (1C; 3-fluoro-4-methoxyphenyl C^5^), 144.3 (1C; 3-fluoro-4-methoxyphenyl C^1^), 152.9 (1C; 3-fluoro-4-methoxyphenyl C^3^), 159.3 (1C; pyridazinone C^6^), 159.4 (1C; 4-methoxyphenyl C^4^), 161.2 (1C; 3-fluoro-4-methoxyphenyl C^4^), 163.2 (1C; CH_2_–N–C=O), 168.0 (1C; pyridazinone C^3^); C_21_H_20_FN_4_O_4_ MS (ESI+) Calcd.: 411.1469, Found: *m*/*z* 411.1483 (M^+^; 100.0%).

##### 2.1.6.9. N’-(4-nitrobenzylidene)-2–(3-(3-fluoro-4-methoxyphenyl)-6-oxopyridazin-1(6H)-yl)acetohydrazide (VIi)

White crystals; yield: 38%; M.P.: 265 °C; IR (ν cm^−1^, ATR): 1705 (C=O; hydrazone), 1647 (C=O; pyridazinone ring), 1581 (C=N); ^1^H-NMR (DMSO-d_6_, 300 MHz): *δ* 3.90 (3H; s; CH_3_O), 5.35 (2H; s; –N–CH_2_–C=O), 7.10 (1H; d; pyridazinone H^5^), 7.26 (1H; d; pyridazinone H^4^), 7.12–8.29 (7H; m; phenyl protons), 8.31 (1H; s; –N=CH–), 12.06 (1H; s; –NH–N); ^13^C-NMR (DMSO-d_6_, 300 MHz): *δ* 53.8 (1C; CH_3_O), 56.4 (1C; –N–CH_2_–C=O), 113.4 (1C; =CH), 113.6 (1C; pyridazinone C^5^), 113.7 (1C; 4-nitrophenyl C^3^), 114.3 (1C; 4-nitrophenyl C^5^), 124.4 (1C; 4-nitrophenyl C^2^), 124.4 (1C; 4-nitrophenyl C^6^), 127.5 (1C; pyridazinone C^4^), 128.3 (1C; 3-fluoro-4-methoxyphenyl C^2^), 130.0 (1C; 3-fluoro-4-methoxyphenyl C^6^), 140.6 (1C; 4-nitrophenyl C^1^), 142.9 (1C; 3-fluoro-4-methoxyphenyl C^5^), 148.2 (1C; 3-fluoro-4-methoxyphenyl C^1^), 151.2 (1C; 3-fluoro-4-methoxyphenyl C^3^), 152.8 (1C; pyridazinone C^6^), 159.3 (1C; 4-nitrophenyl C^4^), 159.4 (1C; 3-fluoro-4-methoxyphenyl C^4^), 164.0 (1C; CH_2_–N–C=O), 168.7 (1C; pyridazinone C^3^); C_20_H_17_FN_5_O_5_ MS (ESI+) Calcd.: 426.1214, Found: *m*/*z* 426.1205 (M^+^; 100.0%).

##### 2.1.6.10. N’-(4-isopropylbenzylidene)-2–(3-(3-fluoro-4-methoxyphenyl)-6-oxopyridazin-1(6H)-yl)acetohydrazide (VIj)

White crystals; yield: 71%; M.P.: 164 °C; IR (ν cm^−1^, ATR): 1700 (C=O; hydrazone), 1656 (C=O; pyridazinone ring), 1584 (C=N); ^1^H-NMR (DMSO-d_6_, 300 MHz): *δ* 1.21 (6H; d; CH_3_), 2.90–2.95 (1H; q; –CH–), 3.90 (3H; s; CH_3_O), 5.28 (2H; s; –N–CH_2_–C=O), 7.09 (1H; d; pyridazinone H^5^), 7.26 (1H; d; pyridazinone H^4^), 7.11–8.13 (7H; m; phenyl protons), 8.20 (1H; s; –N=CH–), 11.71 (1H; s; –NH–N); ^13^C-NMR (DMSO-d_6_, 300 MHz): *δ* 24.1 (2C; CH_3_), 39.6 (1C; CH), 53.9 (1C; CH_3_O), 56.5 (1C; –N–CH_2_–C=O), 114.4 (1C; =CH), 127.1 (1C; pyridazinone C^5^), 127.2 (2C; 4-isopropylphenyl C^3,^5), 127.3 (1C; 4-isopropylphenyl C^4^), 127.4 (2C; 4-isopropylphenyl C^2,^6), 127.5 (1C; pyridazinone C^4^), 132.1 (2C; 3-fluoro-4-methoxyphenyl C^5,^6), 142.8 (1C; 4-isopropylphenyl C^1^), 144.6 (1C; 3-fluoro-4-methoxyphenyl C^2^), 148.6 (1C; 3-fluoro-4-methoxyphenyl C^1^), 151.1 (1C; pyridazinone C^6^), 152.9 (1C; 3-fluoro-4-methoxyphenyl C^3^), 159.3 (1C; 3-fluoro-4-methoxyphenyl C^4^), 159.4 (1C; CH_2_–N–C=O), 168.1 (1C; pyridazinone C^3^); C_23_H_23_FN_4_O_3_ MS (ESI+) Calcd.: 423.1832, Found: *m*/*z* 423.1817 (M^+^; 100.0%).

##### 2.1.6.11. N’-(2-bromobenzylidene)-2–(3-(3-fluoro-4-methoxyphenyl)-6-oxopyridazin-1(6H)-yl)acetohydrazide (VIk)

White crystals; yield: 89%; M.P.: 189 °C; IR (ν cm^−1^, ATR): 1705 (C=O; hydrazone), 1667 (C=O; pyridazinone ring), 1588 (C=N); ^1^H-NMR (DMSO-d_6_, 300 MHz): *δ* 3.89 (3H; s; CH_3_O), 5.32 (2H; s; –N–CH_2_–C=O), 7.09 (1H; d; pyridazinone H^5^), 7.27 (1H; d; pyridazinone H^4^), 7.37–8.38 (7H; m; phenyl protons), 8.58 (1H; s; –N=CH–), 11.95 (1H; s; –NH–N); ^13^C-NMR (DMSO-d_6_, 300 MHz): *δ* 53.9 (1C; CH_3_O), 56.6 (1C; –N–CH_2_–C=O), 113.6 (1C; =CH), 114.3 (1C; pyridazinone C^5^), 123.8 (1C; 2-bromophenyl C^5^), 127.5 (1C; 2-bromophenyl C^3^), 128.4 (1C; 2-bromophenyl C^4^), 130.0 (1C; 2-bromophenyl C^6^), 131.4 (1C; pyridazinone C^4^), 132.2 (2C; 3-fluoro-4-methoxyphenyl C^2,6^), 133.1 (1C; 3-fluoro-4-methoxyphenyl C^5^), 133.5 (1C; 2-bromophenyl C^1^), 142.8 (1C; 3-fluoro-4-methoxyphenyl C^1^), 148.6 (1C; 2-bromophenyl C^2^), 151.2 (2C; 3-fluoro-4-methoxyphenyl C^3^, pyridazinone C^6^), 152.8 (1C; 3-fluoro-4-methoxyphenyl C^4^), 159.4 (1C; CH_2_–N–C=O), 168.4 (1C; pyridazinone C^3^); C_20_H_16_BrFN_4_O_3_ MS (ESI+) Calcd.: 459.0468, Found: *m*/*z* 459.0467 (M^+^; 100.0%).

##### 2.1.6.12. N’-(2-fluorobenzylidene)-2–(3-(3-fluoro-4-methoxyphenyl)-6-oxopyridazin-1(6H)-yl)acetohydrazide (VIl)

White crystals; yield: 72%; M.P.: 224 °C; IR (ν cm^−1^, ATR): 1689 (C=O; hydrazone), 1648 (C=O; pyridazinone ring), 1585 (C=N); ^1^H-NMR (DMSO-d_6_, 300 MHz): *δ* 3.90 (3H; s; CH_3_O), 5.31 (2H; s; –N–CH_2_–C=O), 7.09 (1H; d; pyridazinone H^5^), 7.11 (1H; d; pyridazinone H^4^), 7.26–8.13 (7H; m; phenyl protons), 8.46 (1H; s; –N=CH–), 11.87 (1H; s; –NH–N); ^13^C-NMR (DMSO-d_6_, 300 MHz): *δ* 53.7 (1C; CH_3_O), 56.4 (1C; –N–CH_2_–C=O), 113.4 (1C; =CH), 113.6 (1C; pyridazinone C^5^), 114.3 (1C; 2-fluorophenyl C^5^), 116.3 (1C; 2-fluorophenyl C^3^), 121.9 (1C; 2-bromophenyl C^4^), 126.9 (1C; 2-fluorophenyl C^6^), 127.6 (1C; pyridazinone C^4^), 131.4 (1C; 3-fluoro-4-methoxyphenyl C^2^), 137.4 (1C; 3-fluoro-4-methoxyphenyl C^6^), 142.8 (1C; 3-fluoro-4-methoxyphenyl C^5^), 148.6 (1C; 2-fluorophenyl C^1^), 151.2 (1C; 3-fluoro-4-methoxyphenyl C^1^), 152.9 (1C; 2-fluorophenyl C^2^), 159.4 (1C; 3-fluoro-4-methoxyphenyl C^3^), 160.3 (1C; pyridazinone C^6^), 161.9 (1C; 3-fluoro-4-methoxyphenyl C^4^), 163.6 (1C; CH_2_–N–C=O), 168.4 (1C; pyridazinone C^3^); C_20_H_16_F_2_N_4_O_3_ MS (ESI+) Calcd.: 399.1269, Found: *m*/*z* 399.1257 (M^+^; 100.0%).

##### 2.1.6.13. N’-(4-bromobenzylidene)-2–(3-(3-fluoro-4-methoxyphenyl)-6-oxopyridazin-1(6H)-yl)acetohydrazide (VIm)

White crystals; yield: 69%; M.P.: 256 °C; IR (ν cm^−1^, ATR): 1701 (C=O; hydrazone), 1651 (C=O; pyridazinone ring), 1584 (C=N); ^1^H-NMR (DMSO-d_6_, 300 MHz): *δ* 3.90 (3H; s; CH_3_O), 5.30 (2H; s; –N–CH_2_–C=O), 7.09 (1H; d; pyridazinone H^5^), 7.26 (1H; d; pyridazinone H^4^), 7.11–8.11 (7H; m; phenyl protons), 8.21 (1H; s; –N=CH–), 11.82 (1H; s; –NH–N); ^13^C-NMR (DMSO-d_6_, 300 MHz): *δ* 53.9 (1C; CH_3_O), 56.4 (1C; –N–CH_2_–C=O), 113.4 (1C; =CH), 113.8 (1C; pyridazinone C^5^), 114.3 (1C; 4-bromophenyl C^3^), 114.5 (1C; 4-bromophenyl C^5^), 123.7 (1C; 4-bromophenyl C^2^), 127.5 (1C; 4-bromophenyl C^6^), 129.4 (1C; pyridazinone C^4^), 131.4 (1C; 3-fluoro-4-methoxyphenyl C^2^), 132.3 (1C; 3-fluoro-4-methoxyphenyl C^6^), 133.6 (1C; 4-bromophenyl C^1^), 142.8 (1C; 3-fluoro-4-methoxyphenyl C^5^), 143.4 (1C; 3-fluoro-4-methoxyphenyl C^1^), 148.5 (1C; 3-fluoro-4-methoxyphenyl C^3^), 151.2 (1C; pyridazinone C^6^), 152.9 (1C; 4-bromophenyl C^4^), 159.4 (1C; 3-fluoro-4-methoxyphenyl C^4^), 163.6 (1C; CH_2_–N–C=O), 168.3 (1C; pyridazinone C^3^); C_20_H_16_BrFN_4_O_3_ MS (ESI+) Calcd.: 459.0468, Found: *m*/*z* 459.0446 (M^+^; 100.0%).

##### 2.1.6.14. N’-(4-hydroxybenzylidene)-2–(3-(3-fluoro-4-methoxyphenyl)-6-oxopyridazin-1(6H)-yl)acetohydrazide (VIn)

White crystals; yield: 42%; M.P.: 251 °C; IR (ν cm^−1^, ATR): 1683 (C=O; hydrazone), 1653 (C=O; pyridazinone ring), 1581 (C=N); ^1^H-NMR (DMSO-d_6_, 300 MHz): *δ* 3.90 (3H; s; CH_3_O), 5.25 (2H; s; –N–CH_2_–C=O), 6.81 (1H; d; pyridazinone H^5^), 6.83 (1H; d; pyridazinone H^4^), 7.08–8.12 (7H; m; phenyl protons), 9.92 (1H; s; –N=CH–), 11.55 (1H; s; –NH–N); ^13^C-NMR (DMSO-d_6_, 300 MHz): *δ* 53.8 (1C; CH_3_O), 56.5 (1C; –N–CH_2_–C=O), 113.5 (1C; =CH), 113.7 (1C; pyridazinone C^5^), 114.4 (1C; 4-hydroxyphenyl C^3^), 116.1 (1C; 4-hydroxyphenyl C^5^), 116.1 (1C; 4-hydroxyphenyl C^2^), 122.9 (1C; 4-hydroxyphenyl C^6^), 125.4 (1C; pyridazinone C^4^), 127.6 (1C; 3-fluoro-4-methoxyphenyl C^2^), 129.1 (1C; 3-fluoro-4-methoxyphenyl C^6^), 131.3 (1C; 4-hydroxyphenyl C^1^), 142.8 (1C; 3-fluoro-4-methoxyphenyl C^5^), 144.8 (1C; 3-fluoro-4-methoxyphenyl C^1^), 148.5 (1C; 3-fluoro-4-methoxyphenyl C^3^), 151.2 (1C; pyridazinone C^6^), 152.9 (1C; 4-hydroxyphenyl C^4^), 159.8 (1C; 3-fluoro-4-methoxyphenyl C^4^), 159.9 (1C; CH_2_–N–C=O), 167.9 (1C; pyridazinone C^3^); C_20_H_17_FN_4_O_4_ MS (ESI+) Calcd.: 397.1312, Found: *m*/*z* 397.1309 (M^+^; 100.0%).

##### 2.1.6.15. N’-(2,4-dichlorobenzylidene)-2–(3-(3-fluoro-4-methoxyphenyl)-6-oxopyridazin-1(6H)-yl)acetohydrazide (VIo)

White crystals; yield: 91%; M.P.: 229 °C; IR (ν cm^−1^, ATR): 1705 (C=O; hydrazone), 1645 (C=O; pyridazinone ring), 1582 (C=N); ^1^H-NMR (DMSO-d_6_, 300 MHz): *δ* 3.90 (3H; s; CH_3_O), 5.32 (2H; s; –N–CH_2_–C=O), 7.08 (1H; d; pyridazinone H^5^), 7.11 (1H; d; pyridazinone H^4^), 7.10–8.13 (6H; m; phenyl protons), 8.35 (1H; s; –N=CH–), 11.98 (1H; s; –NH–N); ^13^C-NMR (DMSO-d_6_, 300 MHz): *δ* 53.7 (1C; CH_3_O), 56.7 (1C; –N–CH_2_–C=O), 113.4 (1C; =CH), 113.5 (1C; pyridazinone C^5^), 113.7 (1C; 4-chlorophenyl C^3^), 114.4 (1C; 4-chlorophenyl C^5^), 129.9 (1C; 4-chlorophenyl C^6^), 130.7 (1C; pyridazinone C^4^), 131.4 (1C; 3-fluoro-4-methoxyphenyl C^2^), 134.1 (1C; 3-fluoro-4-methoxyphenyl C^6^), 135.5 (1C; 4-chlorophenyl C^1^), 139.5 (1C; 3-fluoro-4-methoxyphenyl C^5^), 142.9 (1C; 3-fluoro-4-methoxyphenyl C^1^), 148.6 (1C; 3-fluoro-4-methoxyphenyl C^3^), 151.2 (1C; pyridazinone C^6^), 152.8 (1C; 4-chlorophenyl C^2^), 159.3 (1C; 4-chlorophenyl C^4^), 159.4 (1C; 3-fluoro-4-methoxyphenyl C^4^), 163.7 (1C; CH_2_–N–C=O), 168.5 (1C; pyridazinone C^3^); C_20_H_15_Cl_2_FN_4_O_3_ MS (ESI+) Calcd.: 449.0583, Found: *m*/*z* 449.0574 (M^+^; 100.0%).

### 2.2. Pharmacology

#### 2.2.1. Artemia salina lethality test in vivo

In *Artemia salina* lethality bioassay, brine shrimp larvae were incubated for 24 h with compounds **VIa**-**o** (0.01–10 mg/mL) dissolved in the incubation medium (artificial sea water). The detailed protocol was described in our previous article^20^.

#### 2.2.2. Human colon cancer HCT116 cell culture and experiments in vitro

HCT116 cell line (ATCC® CCL-247™) was cultured in DMEM (Euroclone) supplemented with 10% (*v*/*v*) heat-inactivated foetal bovine serum and 1.2% (*v*/*v*) penicillin G/streptomycin in 75 cm^2^ tissue culture flask (*n*=5 individual culture flasks for each condition) as previously reported with or without serotonin treatment^20^.

In the same condition, the kynurenic acid (KA) extracellular level was determined through a validated high performance liquid chromatography (HPLC)-fluorimetric method^21^. To assess the cytotoxicity of synthesised compounds (**VIa–o**), a viability assay was performed on 96 microwell plates, using 3-(4,5-dimethylthiazol-2-yl)-2,5-diphenyltetrazolium bromide (MTT) test. Cells were incubated with compounds (10 µg/mL) for 24 h. An aliquot of 10 µL of MTT (5 mg/mL) was added to each well and incubated for 3 h. The viability of HCT116 cell line was evaluated both in basal conditions and after challenging with serotonin (5-HT) at 1 ng/mL. The anti-proliferative effects were compared to that induced by daunorubicin (0.1–20 µg/mL), used as reference drug.

Finally, the effects of the most potent compounds were evaluated on the spontaneous migration of HCT116 cells, in the 48 h following the experimental lesion of cell monolayer (wound healing paradigm). The detailed protocol related to wound healing experimental model was described in our previous article^20^.

#### 2.2.3. Statistical analysis

Results of *in vitro* studies were expressed as means ± standard error (SE) of three experiments performed in triplicate. Statistical analysis was determined through analysis of variance (ANOVA), followed by *post hoc* Newman-Keuls comparison multiple test. The level of significance was set at *p* < 0.05.

## 3. Results and discussion

**Scheme 1.**
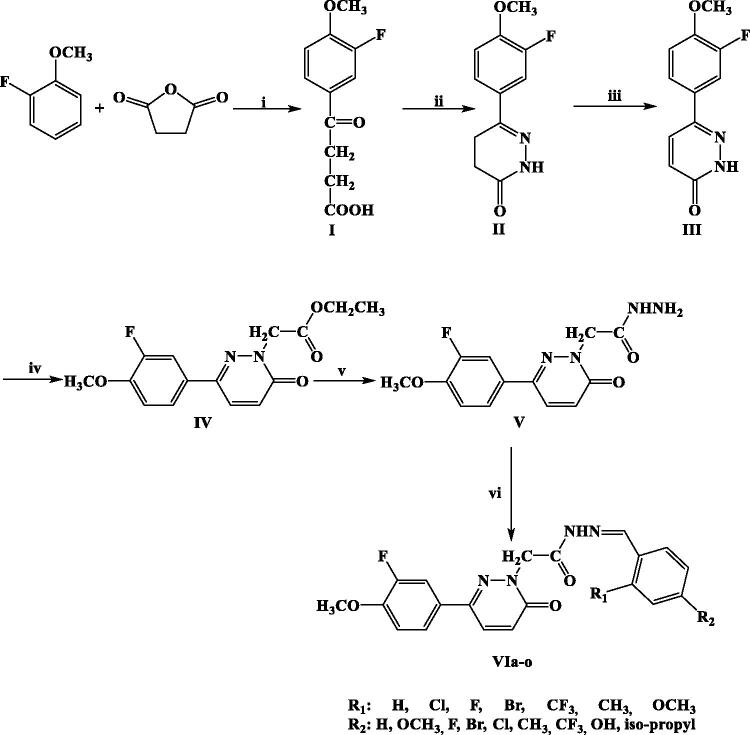
Synthesis of compounds **VIa-o**. Reagents and conditions: (i) AlCl_3_, CS_2_; (ii) H_2_NNH_2_, EtOH, reflux (6 h); (iii) Br_2_, CH_3_COOH, reflux (overnight); (iv) BrCH_2_COOCH_2_CH_3_, K_2_CO_3_, acetone, reflux (overnight); (v) H_2_NNH_2_^.^H_2_O, MeOH, rt; (vi) EtOH, reflux (6 h), nonsubstituted/substitutedbenzaldehyde.

In order to enlarge our SAR on this chemical scaffold, we investigated the presence of different substituents on the hydrazone moiety to explore the chemical space in terms of electronic and steric effects. Moreover, we deleted the piperazine linker attached to the pyridazinone core nucleus aiming at limiting the conformational freedom of our compounds. The title compounds (**VIa–o**) were synthesised according to the literature methods as outlined in Scheme 1.

Synthesis of the compounds was initiated by obtaining benzoyl propanoic acid derivative (**I**) in the presence of succinic anhydride and 2-fluoroanisole by anhydrous aluminium chloride catalysis. Subsequently, the reaction of this compound with hydrazine hydrate led to the formation of 4,5-dihydro-3(*2H*)-pyridazinone (**II**). 6-Substituted-3(*2H*)-pyridazinone derivative (**III**) was obtained by oxidation of **II** with bromine in glacial acetic acid. Ethyl 6-substituted-3(*2H*)-pyridazinone-2-ylacetate derivative (**IV**) was obtained by the reaction of **III** with ethyl bromoacetate in the presence of K_2_CO_3_ in acetone. Then, 6-substituted-3(*2H*)-pyridazinone-2-ylacetohydrazide derivative (**V**) was synthesised by the condensation reaction of **IV** with hydrazine hydrate. Ultimately, the title compounds bearing benzylidenhydrazide structure were obtained by the reaction of **V** with substituted/nonsubstituted benzaldehydes. All of the title compounds were reported for the first time in this study. The reaction yields ranged approximately from 38% to 96%. Compound **VIe** was synthesised with the highest yield (96%), while compound **VIi** with the lowest yield (38%). The physical and spectral properties of the starting compounds were in accordance with the literature. Molecular structures of title compounds were confirmed by IR, ^1^H-NMR, ^13^C-NMR, and mass spectral data. Their molecular structures, yields, and melting points are given in Table 1.

**Table 1.** Molecular structures, yields and melting points of **VIa–o**.


Compound	*R*_1_	*R*_2_	Yield (%)	mp (°C)
**VIa**	H	H	80	132
**VIb**	H	F	83	238
**VIc**	H	CF_3_	84	252
**VId**	CF_3_	H	68	222
**VIe**	CH_3_	H	96	210
**VIf**	OCH_3_	H	80	214
**VIg**	H	CH_3_	74	220
**VIh**	H	OCH_3_	85	207
**VIi**	H	NO_2_	38	265
**VIj**	H	*i*-C_3_H_7_	71	164
**VIk**	Br	H	89	189
**VIl**	F	H	72	224
**VIm**	H	Br	69	256
**VIn**	H	OH	42	251
**VIo**	Cl	Cl	91	229

**Figure 2.**
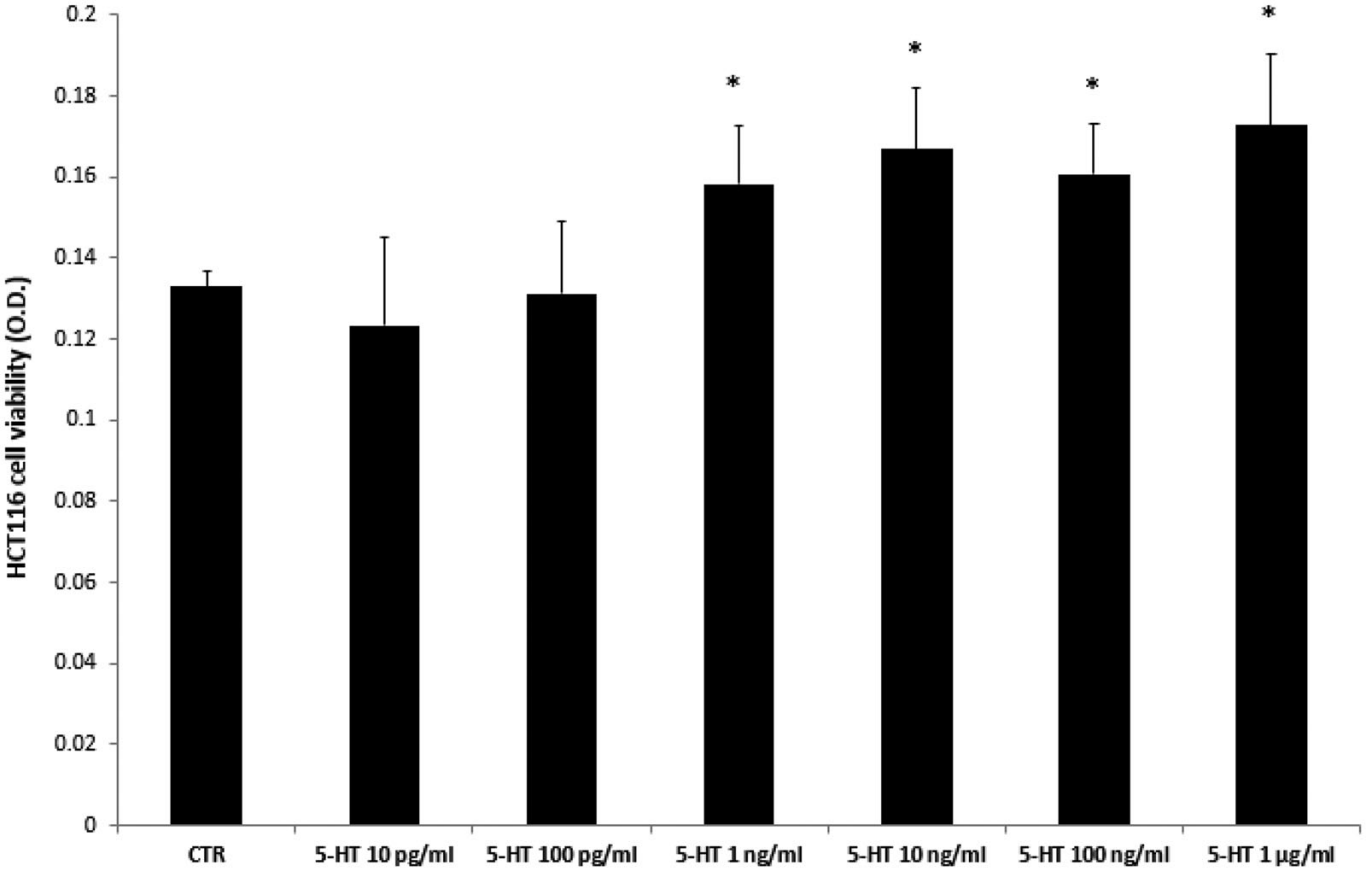
Effects of serotonin (5-HT) in the range 10 pg/mL − 1 µg/mL on colon cancer HCT116 cell viability (MTT test). Data are means ± SE and analysed through analysis of variance (ANOVA), followed by *post hoc* Newman-Keuls test. ANOVA, *p* < 0.01; *post hoc*, *p < 0.05 vs. CTR (control) group.

**Figure 3.**
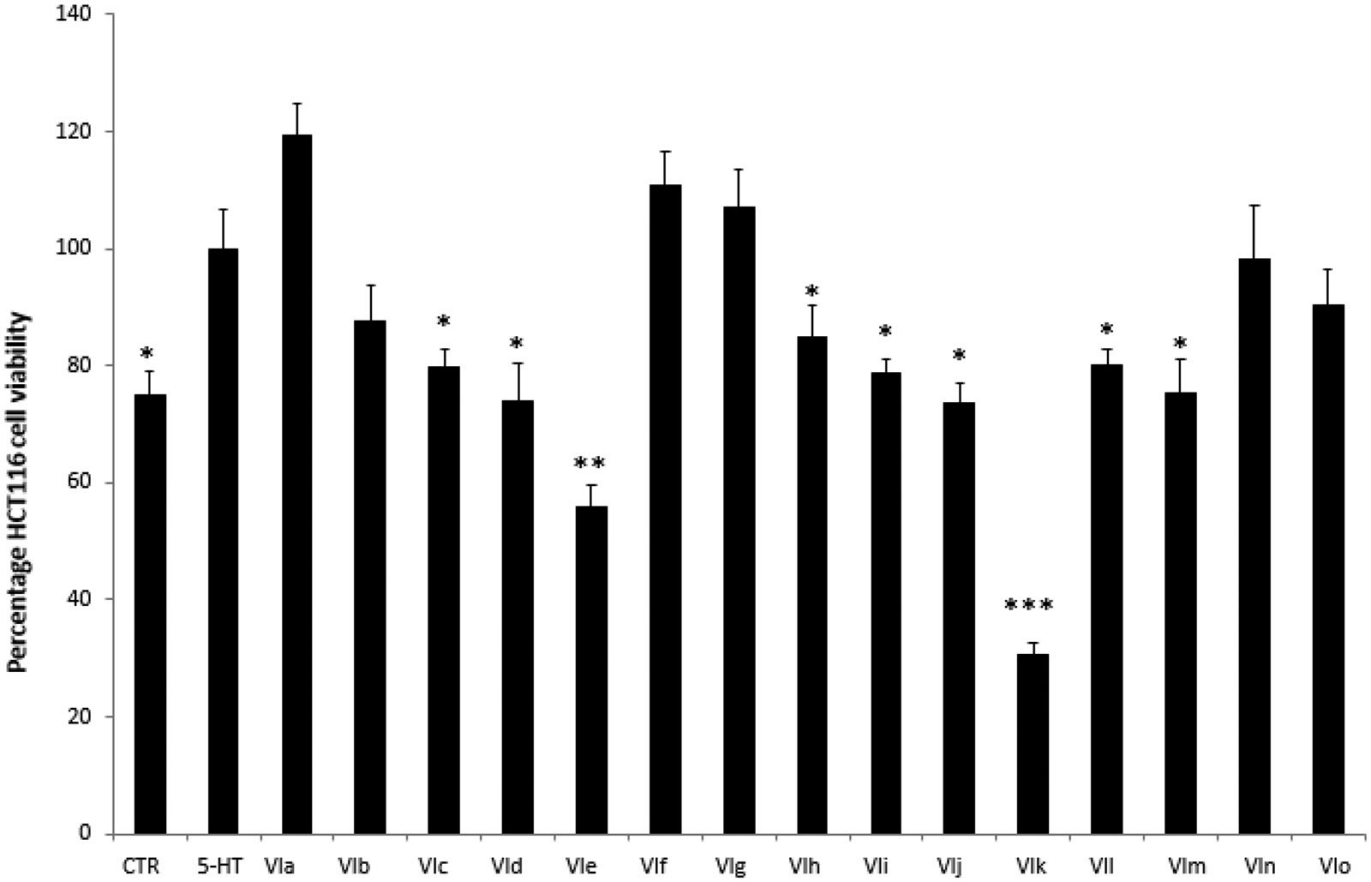
Effects of compounds **VIa**–**o** at 10 µg/mL on serotonin (5-HT)-induced colon cancer HCT116 cell viability (MTT test). Data are means ± SE and analysed through analysis of variance (ANOVA), followed by *post hoc* Newman-Keuls test. ANOVA, *p* < 0.0001; *post hoc*, *p < 0.05, **p < 0.01, ***p < 0.001 vs. 5-HT (serotonin) group.

Firstly, the biocompatibility limit of the compounds was determined through the *A. salina* brine shrimp lethality test *in vivo*. The nauplii were stimulated with compounds **VIa**–**o**, in the range 0.01–10 mg/mL. The lethality test showed LC_50_ values >100 µg/mL for all the compounds. Based on our previous investigations^20^^,^^21^, a 10-fold lower concentration (10 µg/mL) was selected for the subsequent *in vitro* cell-based tests. In this regard, the human colon cancer HCT116 cell line was selected and treated with the aforementioned molecules. The HCT116 viability was stimulated through 5-HT challenging. 5-HT has long been described as a pro-inflammatory factor, particularly in the gut^22^, with *in vitro* studies substantiating a mitogen role, mediated by different receptor types towards multiple cell lines^23^. According to these findings, a preliminary study was carried out in order to optimise the experimental conditions that could demonstrate a cell viability-stimulating effect of 5-HT, in a wide range of concentrations (10 pg/mL – 1 µg/mL). We observed that HCT116 cell viability increased in a concentration-dependent manner, in the range 0.1–1 µg/mL, although it remained constant, at upper tested concentrations (Figure 2) given. Considering our previous *ex vivo* and *in vitro* studies focussed on inflamed colon specimens and hypothalamic cells, respectively, reporting 5-HT concentrations in the order of 1 ng/mL^20^^,^^21^, we have chosen the 5-HT concentration of 1 ng/mL as a reliable proliferative stimulus for the following tests. Specifically, compounds **VIc**–**e** and **VIh**–**m** were able to inhibit 5-HT-stimulated viability of HCT116 cells (Figure 3), thus substantiating the potential anti-proliferative effect of the compounds in the real *in vivo* colon cancer cell microenvironment, characterised by the up-regulated production of multiple pro-inflammatory and anti-apoptotic/mitogen factors, including 5-HT^24^^,^^25^.

**Figure 4.**
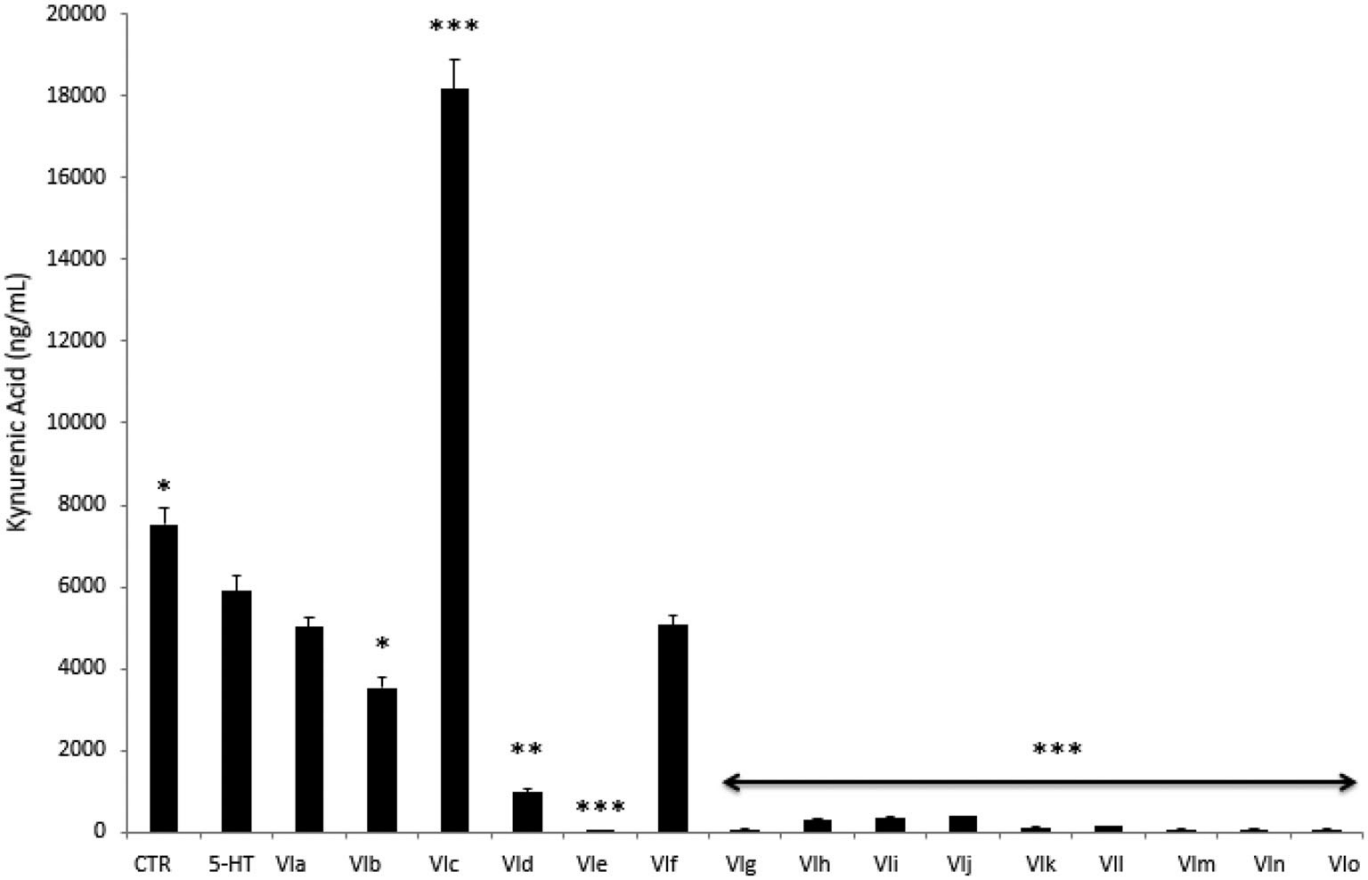
Effects of compounds **VIa–o** at 10 µg/mL on serotonin (5-HT)-induced reduction of kynurenic acid (KA) release from colon cancer HCT116 cells. Data are means ± SE and analysed through analysis of variance (ANOVA), followed by *post hoc* Newman-Keuls test. ANOVA, *p* < 0.0001; *post hoc*, *p < 0.05, **p < 0.01, ***p < 0.001 vs. 5-HT (serotonin) group.

Furthermore, compounds were assayed for evaluating modulatory effects on the extracellular level of KA, one of the two main kynurenine metabolites. Kynurenic acid was reported to be produced in multiple tissues, including brain and peripheral organs^26^, although pharmacokinetic studies excluded any possibility of the peripheral pool to cross blood-brain barrier^27^. In the brain, the kynurenine-derived kynurenic acid was described as a reliable marker of neuroprotection^28^^,^^29^, whereas it seems to be involved in inflammatory response at the peripheral level^30^. Kynurenic acid was also described as an anti-proliferative factor towards colon, renal, and glioma cells^31^. Specifically, this marker was considered as a potential chemopreventive agent against colon cancer^32^^,^^33^. The assessment of KA levels showed that the sole compound **VIc** was able to blunt 5-HT-induced KA depletion (Figure 4) after treatment. Additionally, the KA level after compound **VIc** stimulation was even higher compared to basal condition (CTR group). Conversely, the other tested compounds (**VIa**, **VIb** and **VId**–**m**) failed to prevent the inhibition of KA level following the stimulation with 5-HT. They were able in potentiating 5-HT-induced KA depletion, as well. The results of this pharmacological screening suggest a minor role exerted by the compounds **VIa**, **VIb** and **VId–m** as anti-proliferative agents.

**Figure 5.**
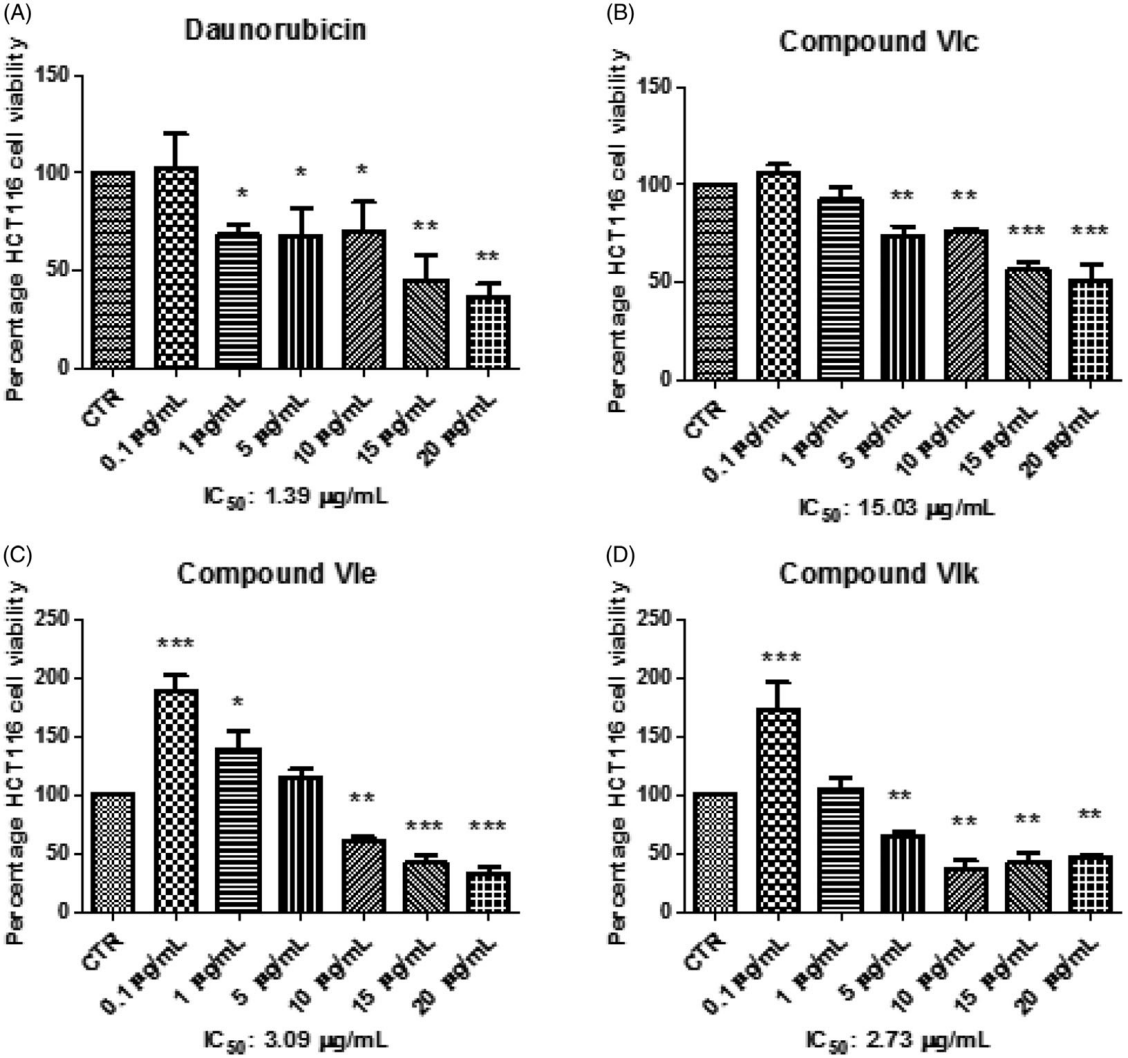
Effects of compounds **VIc**, **VIe**, **VIk** and daunorubicin at 0.1–20 µg/mL on HCT116 cell viability. Data are means ± SE and analysed through analysis of variance (ANOVA), followed by *post hoc* Newman-Keuls test. ANOVA, *p* < 0.0001; *post hoc*, *p < 0.05, **p < 0.01, ***p < 0.001 vs CTR (control) group.

Considering the effects induced by all compounds on HCT116 viability and KA production, compounds **VIc**, **VIe** and **VIk** were further assayed in order to deepen our knowledge about their anti-proliferative effects, in basal conditions. Specifically, HCT116 cells were stimulated with the aforementioned compounds, in a wider concentration range (0.1–20 µg/mL), with respect to the initial test. Additionally, the anti-proliferative effects induced by these compounds were compared with that of daunorubicin, used as anti-tumoral reference drug in the same concentration range. The IC_50_ values were calculated and, as evidenced in Figure 5(A–D), compounds **VIe** and **VIk** showed interesting potencies (Figure 5(C,D)) with IC_50_ values of 3.09 and 2.73 µg/mL respectively, that were very close to that shown by daunorubicin (1.39 µg/mL). Conversely, compound **VIc** showed an IC_50_ value (15.03 µg/mL) that was at least 10-fold higher compared to daunorubicin (Figure 5(A,B)). On the other hand, although the MTT test seems to exclude any application of **VIc** compound as anti-proliferative agent, the increased KA level (Figure 4), shown at a concentration even lower than the IC_50_, indicated a potential use as chemopreventive agent that deserves a further investigation.

**Figure 6.**
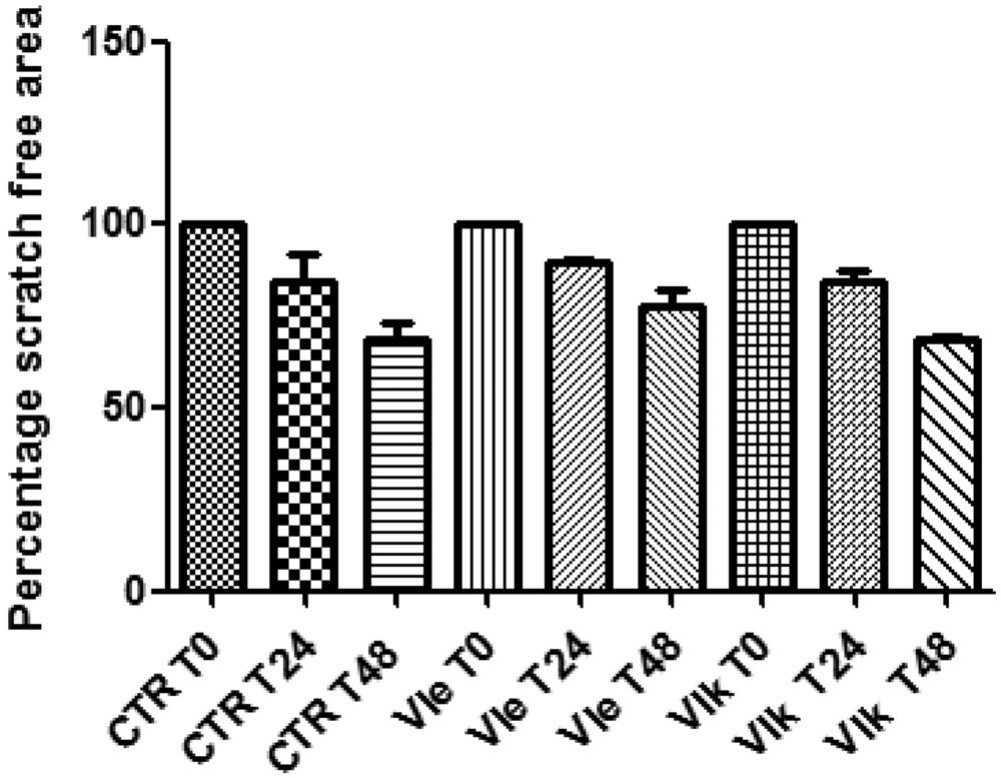
Effects of compounds **VIe** (3.09 µg/mL) and **VIk** (2.73 µg/mL) on the spontaneous migration of human colon cancer HCT116 cell line (wound healing paradigm). The spontaneous migration was monitored in the 48 h following treatment. Data are expressed as percentage scratch area relative to the untreated CTR group.

Finally, considering their potencies in reducing HCT116 cell viability, compounds **VIe** and **VIk** were further studied in order to evaluate their effects on the spontaneous migration of HCT116 cells, through the wound healing paradigm. In this experiment, HCT116 cells were treated with compounds **VIe** and **VIk** at their respective IC_50_ values. The spontaneous migration of HCT116 cells was monitored in the 48 h following the experimental lesion of cell monolayer. The null effects on sponstaneous migration (Figure 6) rule out any involvement of the tested compounds in altering the invasion capacity of human colon cancer cells.

## 4. Conclusion

Fifteen new 3(2*H*)-pyridazinone derivatives were synthesised and studied for their ability to limit the proliferation of HCT116 cell line (colon carcinoma), alone or after stimulation with serotonin, a well-recognized pro-inflamamtory factor in the gut. In particular, compound **VIc** induced a strong release of kynurenic acid after treatment, thus representing a strong chemopreventive agent in this model. Moreover, all compounds resulted non-toxic up to 100 µg/mL in the *A. salina* lethality assay, whereas three of them (**VIc**, **VIe** and **VIk**) displayed a promising inhibitory action comparable to that of daunorubicin as a standard drug at basal conditions.

## References

[1] Vogelstein A, Kinzler KW. The multistep nature of cancer. Trends Genet 1993;9:38–41. 8516849 10.1016/0168-9525(93)90209-z

[2] Smith RA, Andrews KS, Brooks D, et al. Cancer screening in the United States, 2019: a review of current American Cancer Society guidelines and current issues in cancer screening. CA Cancer J Clin 2019;69:184–210. 30875085 10.3322/caac.21557

[3] Ferlay J, Colombet M, Soerjomataram I, et al. Estimating the global cancer incidence and mortality in 2018: GLOBOCAN sources and methods. Int J Cancer 2019;144:1941–53. 30350310 10.1002/ijc.31937

[4] Siegel RL, Miller KD, Jemal A. Cancer statistics, 2020. CA Cancer J Clin 2020;70:7–30. 31912902 10.3322/caac.21590

[5] Banerjee PS. Various biological activities of pyridazinone ring derivatives. Asian J Chem 2011;23:1905–10.

[6] Yamali C, Ozan GH, Kahya B, et al. Synthesis of some 3(2*H*)-pyridazinone and 1(*2H*)-phthalazinone derivatives incorporating aminothiazole moiety and investigation of their antioxidant, acetylcholinesterase, and butyrylcholinesterase inhibitory activities. Med Chem Res 2015;24:1210–17.

[7] Utku S, Gökçe M, Aslan G, et al. Synthesis and in vitro antimycobacterial activities of novel 6-substituted-3(2H)-pyridazinone-2-acetyl-2-(substituted/nonsubstituted acetophenone)hydrazone. Turk J Chem 2011;61:1–9. 10.1055/s-0031-1296161 21355440

[8] Şahina MF, Badıçoglu B, Gökçe M, et al. Synthesis and analgesic and antiinflammatory activity of methyl[6-substituted-3(2H)-pyridazinone-2-yl]acetate derivatives. Arch Pharm Pharm Med 2004;337:445–52. 10.1002/ardp.200400896 15293264

[9] Siddiqui AA, Mishra R, Shaharyar M, et al. Triazole incorporated pyridazinones as a new class of antihypertensive agents: design, synthesis and in vivo screening. Bioorg Med Chem Lett 2011;21:1023–6. 21211966 10.1016/j.bmcl.2010.12.028

[10] Siddiqui AA, Mishra R, Shaharyar M. Synthesis, characterization and antihypertensive activity of pyridazinone derivatives. Eur J Med Chem 2010;45:2283–90. 20189270 10.1016/j.ejmech.2010.02.003

[11] Özçelik AB, Özdemir Z, Sari S, et al. A new series of pyridazinone derivatives as cholinesterases inhibitors: synthesis, in vitro activity and molecular modeling studies. Pharmacol Rep 2019;71:1253–63. 31675671 10.1016/j.pharep.2019.07.006

[12] Nagle P, Pawar Y, Sonawane A, et al. Docking simulation, synthesis and biological evaluation of novel pyridazinone containing thymol as potential antimicrobial agents. Med Chem Res 2014;23:918–26.

[13] Bruel A, Logé C, Tauzia ML, et al. Synthesis and biological evaluation of new 5-benzylated 4-oxo-3,4-dihydro-5H-pyridazino[4,5-b]indoles as PI3Kα inhibitors. Eur J Med Chem 2012;57:225–33. 23063566 10.1016/j.ejmech.2012.09.001

[14] Özdemir Z, Başak-Türkmen N, Ayhan İ, et al. Synthesis of new 6-[4-(2-fluorophenylpiperazine-1-yl)]-3(2H)-pyridazinone-2-acethyl-2-(substitutedbenzal)hydrazone derivatives and evulation of their cytotoxic effects in liver and colon cancer cell line. Pharm Chem J 2019;52:923–9.

[15] Çiftçi O, Özdemir Z, Acar C, et al. The novel synthesized pyridazinone derivates had the antiproliferative and apoptotic effects in SHSY5Y and HEP3B cancer cell line. Lett Org Chem 2018;15:323–31.

[16] Rathish IG, Javed K, Ahmad S, et al. Synthesis and evaluation of anticancer activity of some novel 6-aryl-2-(p-sulfamylphenyl)-pyridazin-3(2H)-ones. Eur J Med Chem 2012;49:304–9. 22305543 10.1016/j.ejmech.2012.01.026

[17] Marconi GD, Carradori S, Ricci A, et al. Kinesin Eg5 targeting inhibitors as a new strategy for gastric adenocarcinoma treatment. Molecules 2019;24:3948. 10.3390/molecules24213948 PMC6864856 31683688

[18] Marconi GD, Gallorini M, Carradori S, et al. The up-regulation of oxidative stress as a potential mechanism of novel MAO-B inhibitors for glioblastoma treatment. Molecules 2019;24:2005. 10.3390/molecules24102005 PMC6572653 31130597

[19] Kuchuk O, Tuccitto A, Citterio D, et al. pH regulators to target the tumor immune microenvironment in human hepatocellular carcinoma. Oncoimmunology 2018;7:e1445452. 29900055 10.1080/2162402X.2018.1445452 PMC5993489

[20] Ferrante C, Recinella L, Ronci M, et al. Multiple pharmacognostic characterization on hemp commercial cultivars: focus on inflorescence water extract activity. Food Chem Toxicol 2019;125:452–61. 30711720 10.1016/j.fct.2019.01.035

[21] Di Giacomo V, Chiavaroli C, Orlando G, et al. Neuroprotective and neuromodulatory effects induced by cannabidiol and cannabigerol in rat Hypo-E22 cells and isolated hypothalamus. Antioxidants 2020;9:71. 10.3390/antiox9010071 PMC7022242 31941059

[22] Regmi SC, Park SY, Ku SK, Kim JA. Serotonin regulates innate immune responses of colon epithelial cells through Nox2-derived reactive oxygen species. Free Radic Biol Med 2014;69:377–89. 24524998 10.1016/j.freeradbiomed.2014.02.003

[23] Ballou Y, Rivas A, Belmont A, et al. 5-HT serotonin receptors modulate mitogenic signaling and impact tumor cell viability. Mol Clin Oncol 2018;9:243–54. 30155245 10.3892/mco.2018.1681 PMC6109681

[24] Curtis JJ, Seymour CB, Mothersill CE. Cell line-specific direct irradiation and bystander responses are influenced by fetal bovine serum serotonin concentrations. Radiat Res 2018;190:262–70. 29963973 10.1667/RR15072.1

[25] Tsai FM, Wu CC, Shyu RY, et al. Tazarotene-induced gene 1 inhibits prostaglandin E2-stimulated HCT116 colon cancer cell growth. J Biomed Sci 2011;18:88. 22126303 10.1186/1423-0127-18-88 PMC3247857

[26] Notarangelo FM, Beggiato S, Schwarcz R. Assessment of prenatal kynurenine metabolism using tissue slices: focus on the neosynthesis of kynurenic acid in mice. Dev Neurosci 2019;41:102–11. 31117076 10.1159/000499736 PMC6732239

[27] Fukui S, Schwarcz R, Rapoport SI, et al. Blood–brain barrier transport of kynurenines: implications for brain synthesis and metabolism. J Neurochem 1991;56:2007–17. 1827495 10.1111/j.1471-4159.1991.tb03460.x

[28] Maddison DC, Giorgini F. The kynurenine pathway and neurodegenerative disease. Semin Cell Dev Biol 2015;40:134–41. 25773161 10.1016/j.semcdb.2015.03.002

[29] Oláh G, Herédi J, Menyhárt A, et al. Unexpected effects of peripherally administered kynurenic acid on cortical spreading depression and related blood-brain barrier permeability. Drug Des Devel Ther 2013;7:981–7. 10.2147/DDDT.S44496 PMC3782408 24068867

[30] Marciniak S, Wnorowski A, Smolińska K, et al. Kynurenic acid protects against thioacetamide-induced liver injury in rats. Anal Cell Pathol (Amst) 2018;2018:1–11. 10.1155/2018/1270483 PMC6171262 30327755

[31] Walczak K, Deneka-Hannemann S, Jarosz B, et al. Kynurenic acid inhibits proliferation and migration of human glioblastoma T98G cells. Pharmacol Rep 2014;66:130–6. 24905318 10.1016/j.pharep.2013.06.007

[32] Walczak K, Turski WA, Rajtar G. Kynurenic acid inhibits colon cancer proliferation in vitro: effects on signaling pathways. Amino Acids 2014;46:2393–401. 25012123 10.1007/s00726-014-1790-3 PMC4168223

[33] Walczak K, Turski WA, Rzeski W. Kynurenic acid enhances expression of p21 Waf1/Cip1 in colon cancer HT-29 cells. Pharmacol Rep 2012;64:745–50. 22814028 10.1016/s1734-1140(12)70870-8

